# Deciphering the role of alternative splicing in neoplastic diseases for immune-oncological therapies

**DOI:** 10.3389/fimmu.2024.1386993

**Published:** 2024-04-26

**Authors:** Marcus Bauer, Chiara-Maria Schöbel, Claudia Wickenhauser, Barbara Seliger, Simon Jasinski-Bergner

**Affiliations:** ^1^ Institute of Pathology, Martin Luther University Halle-Wittenberg, Halle (Saale), Germany; ^2^ Institute for Translational Immunology, Brandenburg Medical School (MHB), Theodor Fontane, Brandenburg an der Havel, Germany; ^3^ Department of Good Manufacturing Practice (GMP) Development & Advanced Therapy Medicinal Products (ATMP) Design, Fraunhofer Institute for Cell Therapy and Immunology (IZI), Leipzig, Germany; ^4^ Institute for Medical Immunology, Medical Faculty, Martin Luther University Halle-Wittenberg, Halle (Saale), Germany

**Keywords:** alternative splicing, HLA-G, PD-L1, PD-1, CTLA-4, immune checkpoints, antibody therapy

## Abstract

Alternative splicing (AS) is an important molecular biological mechanism regulated by complex mechanisms involving a plethora of cis and trans-acting elements. Furthermore, AS is tissue specific and altered in various pathologies, including infectious, inflammatory, and neoplastic diseases. Recently developed immuno-oncological therapies include monoclonal antibodies (mAbs) and chimeric antigen receptor (CAR) T cells targeting, among others, immune checkpoint (ICP) molecules. Despite therapeutic successes have been demonstrated, only a limited number of patients showed long-term benefit from these therapies with tumor entity-related differential response rates were observed. Interestingly, splice variants of common immunotherapeutic targets generated by AS are able to completely escape and/or reduce the efficacy of mAb- and/or CAR-based tumor immunotherapies. Therefore, the analyses of splicing patterns of targeted molecules in tumor specimens prior to therapy might help correct stratification, thereby increasing therapy success by antibody panel selection and antibody dosages. In addition, the expression of certain splicing factors has been linked with the patients’ outcome, thereby highlighting their putative prognostic potential. Outstanding questions are addressed to translate the findings into clinical application. This review article provides an overview of the role of AS in (tumor) diseases, its molecular mechanisms, clinical relevance, and therapy response.

## 1 Introduction

RNA splicing is a fundamentally highly regulated process in the expression of most genes, mediated by the spliceosome removing introns from unspliced pre-RNA to produce mature mRNAs, but also affecting nuclear export, mRNA translation, and quality control (1, 2). The principles of constitutive splicing, including the composition and structure of the spliceosomal complexity, have been recently summarized (3). Its function in eukaryotic cells is to produce numerous transcript variants of mRNAs from an initial single unspliced pre-mRNA, leading to increased protein diversity with elevated structural, functional and in some cases even catalytic complexity. Approximately 95% of human genes show splicing, a molecular process of selecting different splice site combinations (4–6).

### 1.1 Alternative splicing – features and molecular mechanisms

The simple splicing of introns followed by exon ligation is known as constitutive splicing, which occurs for most intron containing genes (7). Apart from this, alternative splicing (AS) provides an additional level of regulation and can dramatically increase the number of resulting splice variants (8). This could be controlled by the strength of the splice sites, which is modulated by cis-acting regulatory elements, like sequences with regulatory potential within the intron and/or exon. For example, homozygous single nucleotide polymorphisms (SNPs) in splicing relevant sites within the non-coding introns can affect the AS and, consequently, the resulting final coding mRNA. In addition, the length of exons/introns and even the expression of molecules involved in the splicing process, like trans-acting factors and RNA-binding proteins (RBPs), have an impact on the resulting splicing pattern (9–11).

On average, there exist 8.8 exons and 7.8 introns per annotated gene. Interestingly, the total length of introns and intergenic DNA on each chromosome is significantly correlated with the size of the chromosome (12). A recent study analyzed 21,106 human genes and identified 14.8% of these genes with evidences of at least one intron retention, usually limited to the untranslated regions (UTRs; (13)).

Next to intron retention, four other mechanisms of AS, namely exon skipping, alternative 5’ splice site, alternative 3’ splice site and coordinated exon splicing, have been identified. The alternative exon splicing leading to exon skipping is inducible and, meanwhile, even employed as a molecular therapeutic strategy for, e.g., duchenne muscular dystrophy (DMD). DMD patients with mutations frequently leading to an early stop codon within exon 51 can be treated with anti-sense oligoribonucleotides, specifically binding to this exon 51. As consequence, the mutated exon 51 and its flanking introns are spliced out, resulting in a mRNA that lacks exon 51 but has a restored reading frame encoding a shortened but at least partially functional dystrophin protein (14).

Alternative 5’ splice site and alternative 3`splice site are less common compared to exon skipping, and both AS mechanisms represent an intermediate state between constitutive and alternative cassette exons. The respective exon has on one site a fixed splice site and on the other site two or more competing splice sites, leading after splicing to different lengths of these regions (15). The fifth mechanism of AS forming “mutually exclusive exons” describes a splicing event with coordinated exon splicing – thus, one exon or one group of exons is retained, while the other exon or group of exons is spliced out. This splicing mechanism is less disruptive to the later protein structure and is frequently found for ion channels and membranous transporters (16, 17). It is noteworthy, that not all resulting splice variants are protein encoding and several protein-coding genes express long non-coding RNA splice variants and can even generate circular RNAs next to their canonical mRNA transcripts (18).

The spliceosome, which does not exist in prokaryotes and archaea and represents one of the most complex molecular machineries catalyzes two transesterification reactions for successful splicing (19). It consists, in general, of five small nuclear RNAs (snRNAs) and over a hundred proteins. So far, two different spliceosomal complexes, the major and the minor spliceosome, can be distinguished, whereby each of them splices its own introns (3, 20). The major spliceosome contains the following five snRNAs: U1, U2, U4, U5, and U6, while the minor spliceosome has the five snRNAs U11, U12, U4atac, U6atac, and U5 (21). Depending on the organism, the major (also named U2-dependent) spliceosome excises approximately 99.5% of introns, while the minor (or U12-dependent) spliceosome excises about 0.5% of introns. Usually only one intron in a gene can be U12-dependent, while the other introns of the same gene are U2-dependently spliced (22). The formation of the spliceosome involves the stepwise assembly of small nuclear ribonucleic proteins (snRNPs), e.g., in the case of the major spliceosome U1, U2, U4/U6, and U5, as well as a large number of non-snRNP proteins (23).

In general, the spliceosome recognizes splicing signals, which are sequence elements located at the 5′ and 3′ of the splice sites, but it remains unclear why certain splice sites are recognized and chosen, while others are not utilized (24). There exists a complex synergy of different cis- acting elements, including the 5′ and 3’ splice site, the polypyrimidine tract, the branchpoint sequence, the exonic/intronic splicing enhancers as well as exonic/intronic splicing silencers (23). In addition, trans-acting factors, such as RBPs, like the serine arginine-rich proteins and the heterogeneous nuclear ribonucleoproteins (hnRNPs), transcript modifications, and RNA secondary structures are contributing to the complex splicing process (25). Furthermore, the different RNA-binding motifs of SR proteins and hnRNP can also indirectly induce or reduce the gene expression of genes, including, among others, ICP molecules. For example, hnRNP C induces p65 and thereby indirectly CD80 and CD40 (26).

In addition, recent research has discovered altered AS in cells exposed to many kinds of stresses, like aging, viral infections, neoplastic transformation, and even chronic inflammation (27–29). Although AS is a key process for increasing the transcriptome and proteomic diversity, there exist a lack in the understanding of the functional consequences of these changes. Furthermore, AS is interconnected with other molecular regulatory processes at the epigenetic, transcriptional, and posttranscriptional levels (30).

The cellular AS also affects microRNAs (MiRs), which are small (~21 nts) non-coding RNAs binding sequence specifically to their target mRNAs, leading to translational inhibition as well as mRNA decay. This binding occurs preferentially, but not exclusively, to the 3’-untranslated region (UTR) (31). Since intron retention in eukaryotes predominantly occurs within the UTRs, but also in the case of microRNA (miR) binding within the coding sequence, splice variant-specific regulation by miRs in the host cell might be possible for various transcripts. In addition, indirect effects of miRs on AS should be considered, for instance, if a splicing regulating factor of the host cell is disbalanced/targeted by miRs, causing alterations to the AS pattern.

Interestingly, even oncogenic viruses like human herpes virus (HHV) and human papilloma virus (HPV) encode for their own miRs, but these molecules and their regulatory potential for AS have not yet been addressed in detail.

Independently of virus-encoded or endogenous human miRs, the context of miRs and AS also includes the human introns, where about 60% of the human endogenous miRs are encoded (32). The down-regulation of classified anti-tumoral acting endogenous miRs, like hsa-miR-34A-5p, or the overexpression of oncogenic miRs, like hsa-miR-21-5p, represent stand-alone molecular mechanisms able to cause malignant transformation (33, 34).

Since splicing profiles of healthy and viral infected or neoplastic transformed cells displaying a high diversity associated with a widely deregulated AS in tumor diseases. Therefore, there is a specific focus on the protein coding splice variants of selected tumor immune checkpoint (ICP) molecules, which frequently exert altered expression and/or altered AS in neoplastic diseases but also already in conditions contributing to oncogenic transformation like oncogenic viral infections or chronic inflammatory diseases.

## 2 Altered alternative splicing in virus-induced and neoplastic diseases

High-throughput analyses revealed that AS regulates and is regulated by many biological processes. Splicing profiles of healthy, virus-infected and/or neoplastic transformed cells display a high diversity associated with a widely deregulated AS in cancer. Therefore, a specific focus is on the protein-coding splice variants of selected tumor immune checkpoint (ICP) molecules, which frequently exert altered expression and/or AS in tumor diseases, but also already in conditions contributing to oncogenic transformation like oncogenic viral infections or chronic inflammation. These features can influence the efficacy of (immune)therapies, and they can provide novel treatment opportunities. The relevance of AS for therapeutic approaches is exemplified and discussed for different immunological (anti-tumoral) therapies. All the above mentioned issues will be addressed in the following parts in much more detail under the aspect of altered AS and its putative role for respective therapies focusing on immune-based targeted oncological therapies, which strongly depend on the structure (for instance, the existence and/or steric accessibility of the respective epitope).

### 2.1 Altered alternative splicing in selected oncogenic viral diseases

Certain viral infections are associated with the development of several tumor types, and such oncoviruses are implicated in approximately 15-20% of all human cancers (35). Oncogenic viruses can convert normal cells into cancer cells by modulating different metabolic pathways, altering genomic integrity mechanisms as well as the expression of immune modulatory molecules, and inhibiting the apoptotic pathways associated with enhanced cell proliferation. So far, seven oncogenic viruses have been identified to promote tumorigenesis. These include the DNA viruses HPV, HBV, EBV, KSHV, MCByV) and the RNA viruses HCV and HTLV-1. For certain viruses, the splicing machinery is highjacked to produced viral proteins and maintain the life cycle of the virus (36). Therefore, it is not surprising that viruses can also affect the molecular processes of AS, leading to the diversification of the viral proteome and an altered splicing pattern within the host cell. This also affects mRNAs of genes with relevance for immune surveillance and immune evasion, indicating co-evolutionary formed molecular interactions between the host and viruses as a result of selective pressure. Indeed, splicing variants of innate but also of adaptive immune response have been shown to exhibit viral defense (2, 36, 37). Even in plant immunity, AS plays an important role due to the generation of specialized splicing variants of plant resistance genes against pathogen infections, such as plant viruses (2). Furthermore, certain viral encoded transcripts can be spliced by the host cell, which is so far known for adenoviruses and cytomegaloviruses (14, 38, 39).

Currently, an increasing number of publications describe different human viruses interfering with AS to prevent immune recognition. These viruses range from ss/dsRNA viruses, like, *inter alia*, Influenza A, Zika, Dengue virus, Lentivirus and also SARS-CoV-2 to dsDNA viruses, like, e.g., human herpes viruses, as well as papilloma viruses (HPV) (40, 41). The high-risk variants of HPV and a γ-subfamily of the human Herpes viruses consisting of Epstein-Barr Virus (EBV, HHV4) as well as Kaposi’s sarcoma-associated herpes virus (KSHV, HHV8) have a strong oncogenic potential (42, 43). This raises the question, whether the virus-driven impact on AS contributes to the molecular processes of malignant transformation. For EBV, the two long non-coding RNAs EBER1 and EBER2, which are expressed even during the different latency phases, have been reported to interfere with AS within the infected host cells (35, 41, 44).

In addition, comparative analyses revealed an impact of the AS process mediated by the Hepatitis B Virus (HBV) and Hepatitis C Virus (HCV) and identified some host cell genes with an altered AS with relevance to carcinogenesis (45) thereby supporting the hypothesis that viral-driven altered AS patterns in the infected host cell contribute to malignant transformation. During disease progression, an increasing number of host cell genes become altered in their expression, associated with an accumulation of disbalanced AS patterns.

### 2.2 Altered alternative splicing in cancer

High-throughput RNA sequencing studies demonstrated that neoplastic diseases are characterized by transcriptome-wide aberrant splicing when compared to normal tissues. In fact, deregulated RNA splicing is a very common molecular feature that exists in almost all tumor types, including solid tumors as well as hematological neoplasms. These splicing abnormalities influence all steps of oncogenesis, which is clearly underlined by disrupting physiological processes, involving different tumor suppressors and oncogenes, promoting cell cycle and the epithelial-to-mesenchymal transition, as well as enhancing cellular metabolism (2, 35, 36).

Comprehensive bioinformatics analysis demonstrated AS signatures that were associated with malignant transformation in kidney tumors by altering metabolic pathways (37). Moreover, AS can increase cell proliferation and can suppress apoptotic pathways in tumor cells. In a very recent study, integrated multi-omics analyses identified CDCA5 as a prognostic biomarker in colon cancer that was significantly associated with AS. AS is involved in the progression and metastatic spread of neoplastic diseases and affects the local tumor microenvironment (TME) thereby regulating the immune cell response (38–40) and enhancing tumor progression (41, 42). It influences the repertoire as well as the frequency and function of different immune cell subsets and is correlated with the tumor mutational burden (TMB), which is linked to immunological features like immune cell subpopulations and ICP expression (43). Altogether, AS is involved in the generation of anti-tumor immune responses as well as immune evasion of malignant cells, which has been recently comprehensively summarized (2, 35, 36, 41). AS is often correlated with a more aggressive tumor phenotype and resistance to chemotherapy (2, 35, 36, 44), targeted therapy, and immunotherapy (45) as shown for a variety of different drugs, including therapeutic antibodies like Rituximab (anti-CD20), Blinatumomab (anti-CD19), or the selective estrogen receptor modulator Tamoxifen (45–48).

Despite the extent to which cancer-related AS drives pathogenesis, disease progression, and resistance to therapy is still under investigation (49), several studies showed that recurrent somatic mutations and altered expression of trans-acting factors governing splicing catalysis and regulation cause AS in neoplastic diseases (2, 49). In order to uncover genetic aberrations that are associated with AS, genomic studies were performed and identified mutations in the RNA splicing machinery or spliceosomal factors with the highest prevalence in hematopoietic malignancies, including all forms of myeloid neoplasms (31, 33, 38), but have also been detected at lower frequency in solid tumors (32, 34, 50, 51). A high number of hot spot mutations were found in components of the spliceosomal complex, such as *SF3B1*, *U2AF1*, *SRSF2*, and *ZRSR2*, that are largely exclusive to each other (31). A summary of the frequency of the genetic alterations in the respective splice factors is provided in **Table 1**.

**Table 1:** Summary of the frequency of mutations in splicing factors in different neoplasias.

	*SF3B1*	*SRSF2*	*U2AF1*	*ZRSR2*	Reference
Hematopoetic tumors					
MDS	12-31%	9-18%	8-11%	8%	(52–55)
MPN	6%	8%	4%	3-7%	(52)
MDS/MPN	9%	22-44%	6%	3%	(55, 56)
AML	5%	12%	4-7%	3%	(54, 56)
CLL	5- 18%				(57)
Cancer					
Breast Cancer	1.8%	0.1%	0.2%	0.2%	(58)
Pancreatic Cancer	4%				(59)
Lung Cancer			3%		(60)
Prostate Cancer	1%		0.5%		(61)
Melanoma					
Uveal melanoma	15-37%				(62, 63)
Leptomeningeal melanoma	33%				(64)
Central nervous system tumors					
Prolactinoma	19%				(65)

Analyzing the impact of genetic splice factor alterations on intracellular signaling revealed an activation of transcription factor c-myc in human and mouse cells by *SF3B1* mutations. These mutations promoted the decay of mRNA transcripts encoding the PP2A subunit PPP2R5A, thereby increasing the phosphorylation of c-myc S62 and BCL2 S70, which supports c-myc protein stability and impairs apoptosis, respectively (66). In contrast, due to *SRSF2* gene mutations, the sequence-specific RNA-binding activity is altered, thereby changing the recognition of specific exonic splicing enhancer motifs and driving recurrent mis-splicing of key cell regulators (67). Concerning *U2AF1* gene mutations, an activation of the transcription factor FOXO3a that mediates a variety of cellular processes, including apoptosis, proliferation, cell cycle progression, and DNA damage has been described (68, 69). Furthermore, mutations in splicing factors have a context-dependent prognostic value (70, 71). Studies in patients with myelodysplastic neoplasms (MDS) demonstrated that patients carrying an *SF3B1* mutation have a significantly better prognosis compared with patients without *SF3B1* mutations (72–74). However, patients with mutations in *SRSF2* and *U2AF1* have been shown to have an adverse outcome in MDS and AML (38, 75–77), while others showed no significant prognostic impact of mutations in *U2AF1*, *ZRSR2*, and *SF3B1* on patients’ outcome (78). Beyond genomic alterations, the expression of different AS-related splice variants has prognostic value. In prostate cancer, the most common male tumor type, transcriptomic analysis of AS revealed prognostic signatures effectively distinguishing high-risk and low risk patients (79), while epigenetic changes and SRSF2 modifications can enhance T cell exhaustion by histone modification of several ICP molecule genes (80).

Furthermore, the hallmark of the cancer “genome instability and mutation” impairs AS pattern, driving the cell towards malignant transformation. So far, several mutations in cancer-related genes have been reported with altered AS, including mutated KRAS, leading to reduced anti-tumoral immune surveillance (46). In addition, mutated p53 has also been reported to alter AS, thereby activating oncogenic RAS signaling (47). Interestingly, AS may also induce malignant transformation by the two p53 negative regulators MDM2 and MDM4 (MDMX), which are characterized by the existence of more than 70 splice variants for MDM2 (48) and 8 for MDM4 (49), some of which are tumor associated due to their ability to downregulate the tumor suppressor p53 more efficiently than other splice variants, underscoring again the relevance of AS for malignant transformation.

In recent years, inflammation has been shown to favor carcinogenesis, malignant transformation, tumor growth, invasion, and metastatic spread (50). This can be due to faulty immune system regulation, dysbiosis, chronic stress, or other environmental factors, which also affect AS (51, 66–72).

Furthermore, obesity (body mass index, BMI ≥30) that is associated with chronic low-grade inflammation is linked to numerous diseases like type 2 diabetes, fatty liver disease, atherosclerosis, and cardiovascular disorders, but also to many tumor types, including colorectal cancer, renal cancer, post-menopausal breast cancer, esophageal adenocarcinoma, thyroid cancer, endometrial cancer, leukemia, and prostate cancer (75). In fact, the insulin receptor, the leptin receptor, as well as the transcriptional coactivator lipin-1 (required for adipocyte differentiation and lipid metabolism) exert mis-splicing in obesity.

## 3 Alternative splicing of immunomodulatory molecules with consequences for immunotherapies

The complex regulation of immunological surveillance is a finely balanced process characterized by the induction of immune response. Furthermore, the existence of immune-privileged tissues such as cytotrophoblasts in the placenta, cornea tissue and endocrine pancreatic islets, in which inflammation must be avoided to prevent irreversible tissue damage followed by functional loss, requires rigorously controlled expression levels of molecules inhibiting immune surveillance and enhancing local limited immune evasion. Frequently, the same molecular mechanisms used for establishing such physiological immune-privileged tissues can be detected as pathophysiological immune evasion strategies of solid and hematopoietic tumors, as well as of many viral, bacterial, and even fungal infectious diseases, and also with relevance of certain inflammatory diseases (81).

### 3.1 Alternative splicing of non-classical HLA class Ib molecules

Inhibition of immunological surveillance in immune-privileged tissues is achieved by various mechanisms, including the down-regulation of classical human leukocyte antigen (HLA) class Ia molecules (HLA-A, -B, -C) leading to reduced antigen presentation to CD8 positive cytotoxic T lymphocytes (CTLs), which is absolutely crucial in the chorion of the placenta to protect the developing fetus with its paternal antigens from maternal immunity. In addition, non-classical HLA class Ib molecules (HLA-G, -E) have a physiologically tightly regulated expression that is restricted to immune-privileged tissues. Their predominant function shifted from antigen presentation to a role as ligand for strong inhibitory receptors present on all immune effector cells, with HLA-G as ligand for the inhibitory receptors ILT2/LILRB1 (monocytes, dendritic cells, macrophages, B cells, T cells, NK cells), ILT4/LILRB2 (monocytes, dendritic cells, macrophages), KIR2DL4 (NK cells, T cells), as well as HLA-E as ligand for the inhibitory receptor NKG2A (NK cells, T cells) (82). Interestingly, HLA-G is the only HLA class Ia/b molecule characterized by the existence of AS. So far, 7 different HLA-G splice variants have been defined, from which HLA-G1 to G4 encode for membranous proteins, while HLA-G5-G7 encode for secreted proteins due to the loss of the transmembrane domain (**Table 2**). Recently, a novel HLA-G splice variant, namely HLA-GΔα1, has been reported. It lacks the peptide binding groove and does not exert any immune inhibitory function on peripheral blood T and NK cells, which strongly underlines the relevance of AS for the generation of diversity (107).

**Table 2:** Summary of known splice variants in anti-tumor therapy relevant ICP molecules.

ICP	Number of known alternative splicing variants	Clinical relevance of splicing variants	Reference
B7-1/CD80	3 (1 membranous full length and 2 soluble variants s1CD80 and s2CD80)	soluble sCD80-varians inhibit T cells	(83)
B7-2/CD86	3 (1 membranous full length and 2 soluble variants sCD86 and CD86ΔTM)	soluble variante sCD86 shows activating functions	(84, 85)
CD40	3 (1 full length, 1 soluble variant and 1 variant with low ligand binding affinity	indirect inhibitory function of soluble CD40	(86, 87)
CD44	2 (standard (CD44s) and variant (CD44v) isoforms)	not determined	(88)
CTLA4	3 (1 membranous full length, 1 membranous without exon 2 (liCTLA4) and 1 secreted variant without exon 3 (sCTLA4); exon 3 encodes transmembrane domain)	indirect inhibitory function of soluble sCTLA4	(89–92)
CD155/PVR	4 (membranous α- and δ-isoform with exon 6, soluble β isoform with partially loss of exon 6, soluble γ isoform with complete loss of exon 6)	NK and T cell inhibition, soluble CD155 with putative impact on monoclonal antibody therapy in analogy to soluble PD-L1 isoforms	(93, 94)
HLA-G	7 (4 membrane-bound (HLA-G1-4) and 3 solube variants (HLA-G5-7); exon 5 encodes transmembrane domain)	NK and T cell inhibition, soluble HLA-G with putative impact on monoclonal antibody therapy in analogy to soluble PD-L1 isoforms	(81)
ILT2/ILT4	in addition to the membranous variants: also soluble ILT2/ILT4 variants detectable	not determined	(95)
LAG3	2 (1 membranous full length and 1 secreted splice variant; exon 7 encodes transmembrane domain)	membranous LAG3 protein inhibits T cells	(96)
PD-1	5 (2 membranous variants: PD-L1 full length, PD-1Deltaex2 and three secreted variants: PD-1Deltaex3, PD-1Deltaex2,3, and PD-1Deltaex2,3,4; exon 3 encodes transmembrane domain)	clinical relevance of secreted variants	(97, 98)
PD-L1	6 (1 membranous full length, 1 membranous delta IgV-like domain (exon3) variant, 4 secreted splice variants: PD-L1-1, PD-L1-3, PD-L1-9; PD-L1-12; exon 5 encodes transmembrane domain)	clinical relevance of secreted variants with correlated disease progression	(99–101)
PD-L2	3 (1 membranous full length, 1 membranous without exon 3 containing Ig constant-like domain and 1 soluble isoform)	indirect inhibitory function of soluble PD-L2	(102, 103)
SLAM6	3 (1 full length with antagonistic effects on T cells, 2 variants with partially (SLAMF6Δ17–65) and completely (SLAMF6Δ18–128) skipped exon 2)	SLAMF6Δ17–65 boosting T-cell Effector Functions	(104)
TIM-3	2 (1 membranous full length and 1 secreted splice variant)	T cell inhibition	(105)
TIGIT	not determined	not determined	–
CEACAM1	12 different splice variants; with 3 secreted isoforms (CEACAM1-4C1, CEACAM1-3, CEACAM1-3C2)	not determined	(106)

The HLA-G1 and –G5 splice variants exert the highest abundancies. Despite soluble HLA-G fulfills its inhibitory functions in the local micromilieu, it can also be detected in the peripheral blood. For example, elevated HLA-G levels were detected in the peripheral blood of pregnant women, which is even stronger in multigravid women compared to primigravid women (108). Furthermore, elevated sHLA-G serum levels can be more commonly detected in patients with many solid and hematopoietic tumor diseases (109). Recently, BND-22 a monoclonal antibody against ILT2, entered clinical studies with promising results in anti-tumor therapy (110). Furthermore, the efficacy and toxicity of HLA-G-specific CAR-T cells are currently being investigated in clinical trials (111).

In addition to the HLA class Ib molecules, other immune checkpoint (ICP) molecules contribute to the integrity of immune-privileged tissues as well as immune evasion strategies in tumors, which correlate with reduced patient survival rates (112, 113).

### 3.2 Alternative splicing and its consequences of the PD1/PD-L1 and CTLA4 axes

A potent inhibitor of costimulation as a second signal for monitoring T cell response is the inhibitory receptor PD-1 expressed on monocytes, dendritic cells, macrophages, B cells, T cells, and NK cells (114). Recent literature reports different PD1 splice variants next to the full length transcript (97). Due to splicing of exon 3, the PD1Δ3 variant is formed, which encodes for a soluble PD1 protein, which is secreted and indeed interferes with proper inhibitory signaling by binding and sterically blocking its ligands PD-L1 and PD-L2 on the potential target cells for any further binding of the actual membraneous PD1 on immune effector cells, thereby leading to their inhibition (competition). Recently, the clinical relevance of this PD1Δ3 splice variant has been reported for non-small cell lung cancer (98). In this context, it is noteworthy that the anti-PD-1 therapeutic mAbs Nivolumab and Pembrolizumab bind PD-1 in exon 2, since PD-1 splice variants lacking exon 2 have been reported. This should be considered absolutely therapy relevant and influences mAb efficacy (**Tables 2**, **3**). Next to AS of PD-1 soluble and membraneous isoforms of its PD-L1 ligand were identified. The soluble/secreted PD-L1 (secPD-L1) deploys its inhibitory functions to potential immune effector cells in complete analogy to soluble HLA-G also within the micromilieu (99). Concerning PD-L2, up to 8 different splice variants have been reported with so far unknown clinical relevance (133) (splice variants detailed listed in **Table 2**).

**Table 3:** Summary of alternative splice variants of ICP molecules and their interaction with therapeutic ICP blockade.

Target	Antibody-binding site	Exon	Consequence	Reference
CTLA4				
Ipilimumab	front β-sheet of CTLA-4 and intersects with the CTLA-4:B7 recognition surface	2	**splice variant liCTLA4 cannot be bound;** sCTLA4 can bind anti-CTLA4 antibodies and consecutive cell damage will not be initiated	(115–117)
Tremelimumab	The side chain atoms of CTLA4K1 and CTLA4K95 and main chain atoms of CTLA4M3, CTLA4Q41, CTLA4M99, CTLA4Y104, CTLA4L106, and CTLA4I108 participate in the formation of hydrogen bonds between CTLA-4 and tremelimumab, whereas CTLA4E97 participates in salt bridge formation.	2		(116, 118)
ILT2				
BND22	D1 and D2 domains of ILT2	1 – 2	–	(110, 119)
LAG-3				
Relatlimab	D1 and D2 domains of LAG3	4	–	(120–122)
PD-1				
Nivolumab	N-loop, FG and BC loops of the IgV domain	2	**splice variants lacking exon 2 have been described that cannot be bound by anti-PD-1 antibodies**	(100, 123)
Pembrolizumab	C′D loop in the sub-interface I contributes predominantly to the binding affinity of permbrolizumab	2		(100, 124)
PD-L1				
Atezolizumab	interaction between PD-L1 and atezolizumab is mediated largely by residues within the central CC′FG β-sheet of PD-L1 and the heavy chain of atezolizumab	2	sPD-L1 can bind anti-PD-L1 antibody and consecutive cell damage will not be initiated	(100, 125, 126)
Avelumab	The avelumab-binding epitope region on hPD-L1 is predominantly constituted by the C strand, C′ strand, F strand, G strand and CC′ loop of hPD-L1	2		(100, 126, 127)
Durvalumab	Most of the key interactions of PD-L1 with durvalumab are concentrated on the central CC′FG β-sheet within PD-L1	2		(100, 125, 126)
BMS-936559	The side-chain atoms of PDL1D49, PDL1Y56 and PDL1H69 and main-chain atom of PDL1A121 participate in the formation of hydrogen bonds between PD-L1 and BMS-936559, while a salt bridge is formed by PDL1E58	2		(100, 118, 126)
TIGIT				
Vibostolimab	extracellular immunoglobulin variable region	2	–	(128–130)
Tiragolumab	extracellular immunoglobulin variable region	2	–	(129–131)
CEACAM1				
MRG1	N-terminal IgV domain (present on all 12 splice variants)	2	–	(132)

Next to PD1/PD-L1, AS also has relevance for the CTLA4 ICP axis. The receptor CTLA4 on T cells mediates an inhibitory signaling in combination with the ligands B7-1/CD80 and B7-2/CD86 mainly expressed by professional antigen presenting cells (APCs). CTLA4 was the first ICP, which was targeted by mABs and implemented in anti-tumor therapies. Next to the membraneous full length CTLA4, at least 2 different splice variants exist – one variant with ligand independent liCTLA4, lacking exon 2, and one soluble sCTLA4 variant lacking exon 3 (89, 90). Since the anti-CTLA4 mAbs Ipilimumab and Tremelimumab bind CTLA4 in exon 2, they could not inhibit the liCTLA4 splice variant, which is considered as therapy relevant (**Tables 2**, **3**). Regarding CD80, two soluble splice variants can be generated, namely s1CD80 and s2CD80, which both exert inhibitory signaling and functions analogous to soluble HLA-G and PD-L1 (83). AS of CD86 can lead to the soluble splice variant sCD86 lacking the transmembrane domain/exon 6 (CD86ΔTM) and to another membranous splice variant B7-2C (84). Recently, the anti-CD80 antibody Galiximab entered clinical trials [**Table 3** (134)].

### 3.3 Alternative splicing also affects other immune checkpoints, such as LAG3, TIGIT, and CEACAM1

AS occurs also in the LAG-3/CD223 ICP axis. In addition to the membraneous full length LAG3 transcript, one soluble splice variant generated by AS has been reported, but no information about a possible clinical relevance exists (135) (**Table 2**). LAG3 binds to HLA class II molecules of APCs with a higher affinity than CD4 and leads to decreased cell proliferation and activity of these immune effector cells, thus providing another mechanism of immune evasion. Furthermore, tumor cells, e.g., melanoma cells, can also express HLA class II molecules, which was correlated with poorer survival of patients (136). LAG3 blockade using therapeutic mAb, e.g., Relatlimab, is currently being implemented in clinical trials for the treatment of cancer patients, frequently in combination with Nivolumab in melanoma patients (137).

The ICP molecule TIM-3 exhibits one additional soluble splice variant (105). Regarding TIGIT, an inhibitory receptor on NK and T cells, and its ligand CD155/PVR four CD155 splicing variants have been identified with the membranous α and δ isoforms containing the transmembrane domain (exon 6). The δ isoform has an enlarged exon 6 due to additional nucleotides at the end of exon 6. These additional nucleotides of the δ isoform encode for an early stop codon, which leaves exon 7 and 8 untranslated. The soluble β isoform partially lacks exon 6, while the soluble γ isoform has a complete exon 6 loss (93, 94). Currently, the three therapeutic mAbs Vibostolimab, Etigilimab, and Tiragolumab blocking the TIGIT ligand interactions have entered clinical studies with promising results, as recently reviewed by Rousseau and co-authors (138).

Furthermore, CEACAM1 (CD66a) has been identified as an ICP molecule interfering with T, NK, and B cell functions. AS generates 12 different CEACAM1 splice variants, including 3 secreted splice variants lacking the transmembrane domane (CEACAM1-4C1, CEACAM1-3, CEACAM1-3C2). The anti-CEACAM1 therapeutic mAb MRG1 binds to the N-terminal IgV domain of CEACAM1 protein, which is present in all 12 splice variants (106, 132). No clinical relevance of AS of CEACAM1 for the respective antibody therapy has been reported, despite a putative influence of the secreted isoforms is likely.

A summary of putative anti-tumor therapy relevant splicing variants in ICP molecules is listed in **Table 2** and clinically relevant mAb as ICP inhibitors, including their epitopes, are summarized in **Table 3**.

### 3.4 Reconsidering the impact of alternative splicing on immune checkpoint therapies

**Figure 1:**
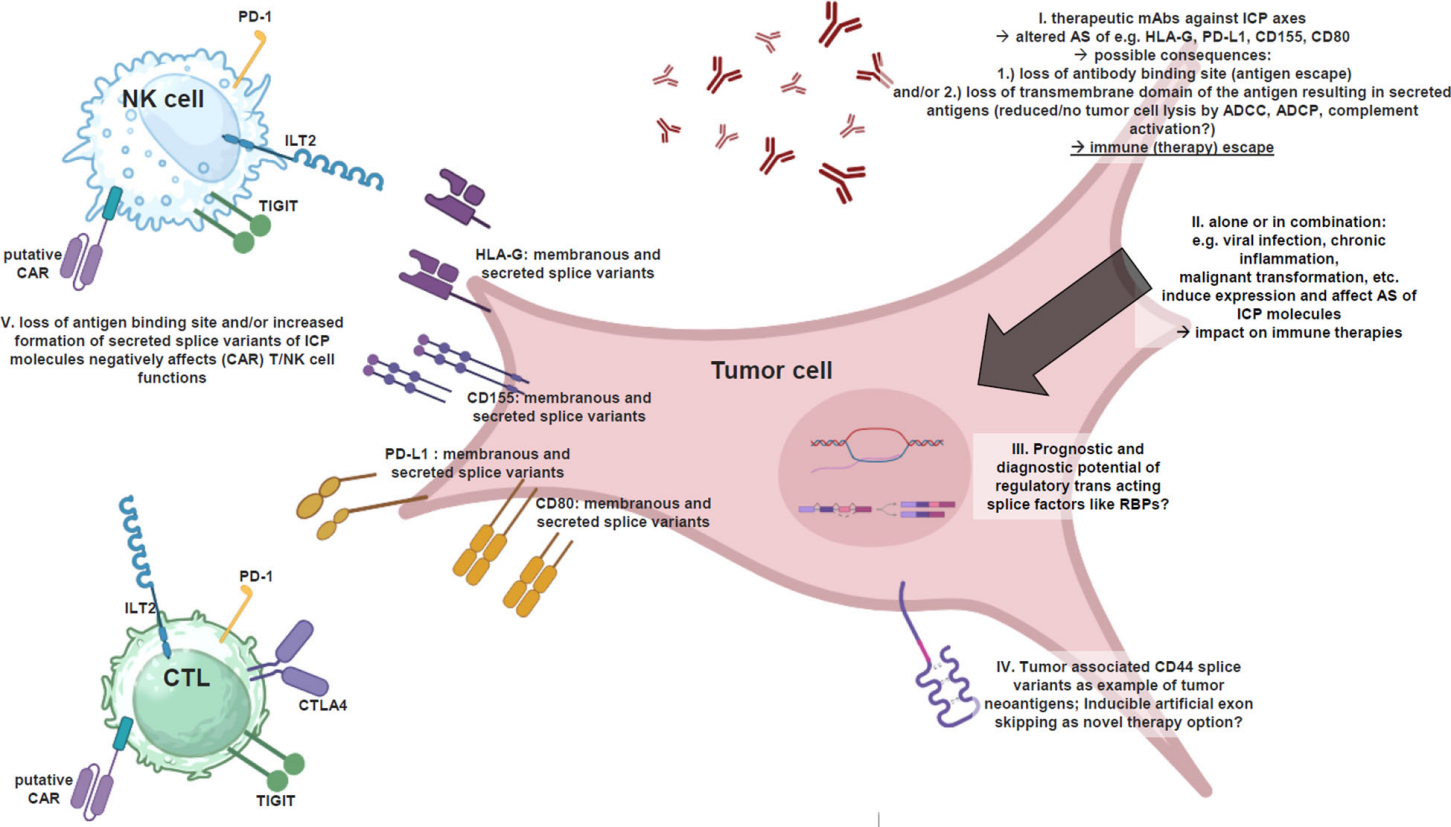
Alternatively spliced ICP ligands interfere tumor immunotherapies. Illustrated summary of alternatively spliced ICP ligands and their impact on anti-tumor immunotherapies based on mAbs and CARs, as well as thought-provoking impulses to implement splicing factors in clinical prognostics and diagnostics for optimal therapy success, as well as artificial exon skipping as a strategy to avoid the formation of secreted ICP ligands and to increase anti-tumor (therapy) immune response (created with BioRender.com). I. The efficacy of therapeutic mAbs against certain ICP axes (e.g. HLA-G, PD-L1, CD155, CD80) may be affected by AS of these molecules due to 1.) loss of antibody binding site (antigen escape) and/or 2.) loss of transmembrane domain of the antigen resulting in secreted antigens causing a reduced or no further tumor cell lysis by ADCC, ADCP, complement activation. II. The AS per se is affected by viral infection, chronic inflammation, malignant transformation, etc. biological processes, which interestingly also induce the expression of certain ICP molecules. III. Is there any prognostic and diagnostic potential of regulatory trans-acting splice factors like RBPs in tumor biopsies prior mAb/CAR therapy selection? IV. Tumor-associated CD44V splice variants as an example for AS based generation of tumor neoantigens serving for novel targets of CARs and therapeutic mAbs. A mechanism, which can even be induced in analogy to exon skipping. V. Putative CARs directed against antigens upon tumor cells (e.g., ICP ligands) are affected by altered AS of targeted antigens in/upon tumor cells, which could lead to 1.) loss of antibody binding site and/or 2.) loss of transmembrane domain of the antigen, resulting in secreted antigens, which will bind to CARs spatially remote from tumor cells ➔ steric blockage of the CARs ➔ no further cell lysis. In addition: secreted ICP ligands inactivate (CAR) T/NK cells already in the tumor microenvironment as immune (therapy) escape.

Due to the fact that an increasing number of mAb based therapies against the above mentioned ICP axes have already successfully received or will receive clinical approval, the expression of at least secreted ICP ligand variants in the targeted tumor tissues should be analyzed as crucial therapy relevant markers prior to therapy selection. This is currently not the case, but the costs of the respective (tumor) immunotherapies with mAbs or CARs are by far higher than any analyses of the targeted ICP molecule splice variants. Tumors with higher levels of secreted ICP ligands and lower expression levels of membranous ICP ligands will certainly be targeted worse by equal amounts of applied therapeutic ICP ligand-binding mAbs, than in *vice versa* tumors, which is an important aspect of the need for cost reduction, but also optimization of treatment efficacy. It should be considered that a single splicing factor potentially leading to an increased splicing of one secreted ICP ligand is also able to increase the splicing of even more co-expressed ICP ligands of other ICP axes with additive effects for (tumor) immune evasion (**Figure 1**) highlighting the potential relevant splice factors of ICP molecules as putative prognostic markers with relevance for therapy selection. Recently, two splicing relevant RNA binding proteins, DHX9 and hnRNPM, have been reported to exert prognostic relevance for ICP inhibitor therapy in non-small cell lung cancer patients (139).

The AS also affects other tumor-relevant molecules apart from the ICPs, which are not directly associated with the efficacy of ICPi therapy. CD44 is a non-kinase cell surface transmembrane glycoprotein that has been shown to play an important role in malignant transformation and tumor progression (88). The full length CD44 gene comprises 20 exons. However, the CD44 gene regularly undergoes AS, resulting in the standard (CD44s) and variant (CD44v) isoforms (88). The smaller and most widely expressed CD44s consists of ten constant exons with no variant exons. CD44v differs from CD44s by the insertion or excision of alternatively spliced exons between the N-terminal and C-terminal domains (140, 141). Hyaluronan is the major ligand of CD44, which activates CD44 and results in the activation of cell signaling pathways that influence cell proliferation and enhance cellular motility (142). Although, the different functional roles of CD44s and specific CD44v isoforms are not completely understood, an important role of CD44v in malignancies has been shown. CD44v is more frequently expressed in metastasized tumors, whereas switching between CD44v and CD44s may play a role in regulating epithelial to mesenchymal transition (142). Moreover, CD44 monitors changes in the extracellular matrix in general, and CD44v influences cell growth, survival, and differentiation (143). CD44v contains additional peptide motifs that can interact with growth factors and cytokines at the cell surface and passing these signals through association with the actin cytoskeleton, thereby functioning as coreceptors to facilitate cell signaling (142, 143). Due to these changes in intra- and extracellular signaling an impaired chemosensitivity has been demonstrated and can be enhanced by knockdown of CD44 in acute myeloid leukemia (AML) cells (144). In addition, CD44s has an independent prognostic significance in hematological neoplasms and can serve as a marker for tumor diagnostics as well as a molecular anti-tumor target for mAb-based therapies or as a target for chimeric antigen receptors (CARs) (145).

Thus, the AS of CD44 is a valuable example addressing the complexity of the role of AS in targeted immunotherapies. In contrast to the AS-mediated reduction in the efficacy of anti-tumor therapies targeting these ICP splice variants, the AS of CD44 creates novel molecular targets, which can be used for novel anti-tumor immunotherapies.

## 4 Perspectives

### 4.1 Alternative splicing and its clinical relevance

Deciphering the clinical role of AS is a very current focus of research, which offers on the one hand the basis for novel (immuno) therapies or therapeutic approaches in general, but on the other hand, it must be noticed that AS creates various splice variants of the same targeted molecule, and the balance between such possible splice variants might be dramatically varying within patients suffering from the same (tumor) disease, and that these diverging splice variants also might be targeted differentially effective by the same therapeutics, for instance by mAbs or CARs.

This point raises several important questions, whether the splicing pattern of targeted molecules should be analyzed before a panel of therapeutic antibodies or a certain CAR can be selected, in dependency of, whether the targeted epitope is present in the most abundant splice variants within the respective (tumor) tissue, which also has relevance for anti-tumor vaccination. Subsequently, the next question follows, even if the epitope is present in the most expressed splice variants of the targeted molecule, will the presence of predominantly secreted splice variants of the targeted molecule require higher (expensive) antibody doses or *vice versa* lower antibody doses if predominantly membranous splice variants are expressed? In fact, secreted ICP ligands will bind their inhibitory receptors upon immune effector cells, and also to the CAR upon the CAR T/NK cells, or the therapeutic mAbs as ICP inhibitors, even before these therapeutics can reach the tumor cell and could induce cell lysis. Without antibody opsonization of the targeted (tumor) cell no antibody-dependent cellular cytotoxicity (ADCC) and phagocytosis (ADCP), and no complement activation can be achieved (**Figure 1**).

Moreover, it should be analyzed whether AS alters the expression variants of targeted molecules, e.g., ICPs as a mechanism of ICP blockade resistance. Introducing an artificial T cell receptor in (autologous) expanded and activated CTLs/NK cells would allow their redirection towards the novel (tumor) antigen before their injection into the patient. Predominantly, CAR T cell therapies exert remarkable clinical results in certain hematological malignancies, like B cell leukemia and lymphoma, using, e.g., CD19 as target antigen, which was the first CAR T cell therapy approved by the US Food and Drug Administration (FDA) in 2017 (146). Despite this success, 50% of patients relapse due to immune rejection and T cell exhaustion, or epitope loss (147).

However, for CAR T cell therapy targeting different antigens and ICPs, relevant splicing events have also been reported. For example, an insufficient activity of the splicing factor SRSF3 has been linked to the abundance of the CD19 Δex2 isoform in relapsed B cell leukemias after CAR T cell therapy leading to resistance (148). In addition, AS driven CAR T cell resistance was also found for CD22 due to skipping of exons 5 and 6 and exon 2 leading to a deregulated splicing (149). Certain limitations of CAR based anti-tumor therapies exist, such as on-target off-tumor effects, antigen escape, low penetration in solid tumors, and an inhibitory micromilieu of solid tumors of injected CAR T cells (146), while the 5’-UTR reduces the transduction of CD20 mRNA, resulting in resistance to CD20-based immunotherapies (150). However, it should be considered that the AS of such ligands can generate secreted soluble isoforms, which are able to block the CAR and inhibit the CAR T/NK cell activity before they even reach the tumor cell. Furthermore, the production of soluble and secreted ICP ligands can also act as an antigen escape mechanism. Tumor cells secreting the antigen into the micromilieu instead of expressing it upon their cell surface, will not be targeted by the CAR T/NK cells anymore.

The next point, which should be addressed in more detail, are the splicing mechanisms of the targeted molecules, whether there are any trans-acting factors like RBPs involved, which could also be of importance as putative prognostic or therapy relevant marker genes, or even get targeted themselves by respective inhibitors in combined therapies to increase the response rates of the (even more expensive) mAbs/CAR therapies. However, the development of inhibitors targeting RBPs or even SFs have been challenging due to the lack of targetable catalytically active sites with the exception of aryl sulfonamides (151).

As mentioned in the introduction, the artificial exon skipping of mutated exon 51 in DMD patients offers a complete new level in the treatment of certain human genetic diseases, already with progressed stages. In the case of exon skipping in DMD patients, various therapeutics have been approved by the FDA, and meanwhile also other mutated exons can be targeted by the same technique in DMD patients. Next to this, also the applied antisense oligonucleotides have been improved over the years, newer ones enhance exon skipping levels more than 100 times when compared to the old ones, strongly underlining the future prospects of this approach (152). Based on this, the authors hypothesize that this technique could also be used in future anti-tumor therapies to induce unusual splice variants upon the tumor cells as putative tumor neoantigens, which could be targeted by respective mAbs/CARs. Next to the targeting of alternative splice factors, several other approaches exist. These include (i) targeting of upstream regulatory proteins, such as protein arginine methyltransferase, known as regulators of both constitutive and AS, (ii) targeted splicing corrections by small molecules targeting individual isoforms, (iii) splicing modulation with oligonucelotides or (iv) gene editing by CRISPR-based strategies targeting specific AS events.

Meanwhile, many different mAb/CAR therapies have already entered clinics targeting various tumor biology relevant molecules even apart from the highlighted ICP axes, e.g., to interfere with neovascularization or the binding of growth factors, alone or in combination with cytokines, cell cycle/mTOR inhibitors, chemotherapeutics, etc.

At this point, it should also be considered to investigate the impact of the above mentioned combined pharmaceuticals on the splicing pattern within the treated tumor. Are such theoretical side effects relevant for the loss of epitope or loss of transmembrane domain in the targeted molecules and thereby relevant for the expensive mAb/CAR therapy?

## Author contributions

MB: Data curation, Formal analysis, Investigation, Methodology, Software, Writing – original draft, Writing – review & editing. CS: Data curation, Formal analysis, Investigation, Writing – original draft. CW: Writing – review & editing. BS: Formal analysis, Investigation, Resources, Writing – review & editing. SJ: Conceptualization, Data curation, Formal analysis, Funding acquisition, Investigation, Methodology, Project administration, Resources, Supervision, Validation, Visualization, Writing – original draft, Writing – review & editing.
